# A Predictive Model to Classify Undifferentiated Fever Cases Based on Twenty-Four-Hour Continuous Tympanic Temperature Recording

**DOI:** 10.1155/2017/5707162

**Published:** 2017-11-22

**Authors:** Pradeepa H. Dakappa, Keerthana Prasad, Sathish B. Rao, Ganaraja Bolumbu, Gopalkrishna K. Bhat, Chakrapani Mahabala

**Affiliations:** ^1^Department of Internal Medicine, Kasturba Medical College, Manipal University, Mangaluru, Karnataka, India; ^2^School of Information Sciences, Manipal Institute of Technology, Manipal University, Manipal, Karnataka, India; ^3^Department of Physiology, Kasturba Medical College, Manipal University, Mangaluru, Karnataka, India; ^4^Department of Microbiology, Kasturba Medical College, Manipal University, Mangaluru, Karnataka, India

## Abstract

Diagnosis of undifferentiated fever is a major challenging task to the physician which often remains undiagnosed and delays the treatment. The aim of the study was to record and analyze a 24-hour continuous tympanic temperature and evaluate its utility in the diagnosis of undifferentiated fevers. This was an observational study conducted in the Kasturba Medical College and Hospitals, Mangaluru, India. A total of ninety-six (*n* = 96) patients were presented with undifferentiated fever. Their tympanic temperature was recorded continuously for 24 hours. Temperature data were preprocessed and various signal characteristic features were extracted and trained in classification machine learning algorithms using MATLAB software. The quadratic support vector machine algorithm yielded an overall accuracy of 71.9% in differentiating the fevers into four major categories, namely, tuberculosis, intracellular bacterial infections, dengue fever, and noninfectious diseases. The area under ROC curve for tuberculosis, intracellular bacterial infections, dengue fever, and noninfectious diseases was found to be 0.961, 0.801, 0.815, and 0.818, respectively. Good agreement was observed [kappa = 0.618 (*p* < 0.001, 95% CI (0.498–0.737))] between the actual diagnosis of cases and the quadratic support vector machine learning algorithm. The 24-hour continuous tympanic temperature recording with supervised machine learning algorithm appears to be a promising noninvasive and reliable diagnostic tool.

## 1. Introduction

Undifferentiated fever is a commonly encountered febrile illness without any localized signs or symptoms [1]. According to a systematic review, the percentage of undiagnosed cases of undifferentiated fever in Asia is about 8% to 80% [2]. In resource-limited countries, the decision regarding clinical investigations at an early stage is a challenging task for the physicians [3]. The nonspecificity of symptoms and lack of availability of accurate diagnosis not only has a significant impact on clinical decision-making but often leads to the irrational use of antibiotics [3, 4]. In most of the undifferentiated fever cases, empirical treatment either does not work or may be harmful and might delay hospitalization of the patient, with subsequent increase in medical expenses.

Monitoring of the fever can provide valuable information for diagnosis and prognosis of the disease. Many scientific studies reported on the utility of temperature monitoring as a predictive tool for certain clinical diseases [5–15]. One century earlier, Woodhead et al. studied the 24–48 hours of quasicontinuous temperature recordings in patients for the diagnosis and prognosis of tuberculosis. In cases of tuberculosis, they observed a few characteristic features of temperature curve like sudden rise of afternoon and evening temperature, rapid fall, continuous high temperature above 99°C for 8 to 9 hours, and mountain peaks on plateau phase [11]. However, not enough studies were done to explore the utility of temperature, probably because of limited hardware capabilities, with cumbersome recording methods and software issues, which were not well developed at that time. Two decades earlier, interest in 24-hour temperature recording system re-emerged after Varela et al. showed continuous recording of body temperature using tympanic and axillary probes and analysis of temperature data [4, 12]. The quantitative measurement of body temperature has shown promising results in the management of hypovolemia, mortality in critically ill patients, diagnosis of lactic acidosis, the prognosis of organ hypoperfusion and shock, besides acting as a marker of cardiovascular status, dyspnea, and tissue perfusion [5–10]. Another study reported that the abnormal body temperature could act as a predictor of the diagnosis of sepsis in febrile, critically ill patients [16]. However, these studies did not address the underlying issue of diagnostic utility of temperature recordings in undifferentiated fevers.

Body temperature is a physiological signal which has essential features and trends associated with it. However, some of this information like minute variations, trends, and patterns in time series domain may not be apparent with conventional methods and may require complex mathematical models for their analysis. Unlike other vital signals like ECG, EEG, and EMG, there are only a limited number of studies on the temperature signal for predicting certain diseases by using mathematical models [12, 13, 15]. Researchers observed the body temperature variations in patients either visually or by using specific mathematical models. Papaioannou et al. studied the temperature patterns using linear discriminant analysis and cluster analysis by extracting wavelet features for the differentiation of patients with systemic inflammatory response syndrome, sepsis, and septic shock. Researchers extracted different wavelet features from the temperature pattern among the three groups (systemic inflammatory response syndrome, sepsis, and septic shock) and found statistically significant outcome [15]. Varela et al. applied approximate entropy and detrended fluctuation analysis (DFA) methods for determining the loss of complexity of the temperature curve associated with the diseased state. They compared results with conventional Sequential Organ Failure Assessment (SOFA) score and found that the temperature curve complexity is inversely related to the severity of patient's status. The approximate values were significantly low in nonsurvivors than in survival patients [13, 17]. In another study, Varela et al. used approximate entropy as a feature and found 72% accuracy in classifying two groups: death and survival patients with multiple organ failure [18]. Two more scientific studies reported the predictive model for differentiating dengue fever cases with other febrile illness, early phase of illness using multivariate logistic regression model and decision tree algorithm [19, 20]. Although these studies were done either in critical care settings for prognostication or for studying the extent of complications, they have not been studied in formal settings of diagnostic utility in undifferentiated tropical fevers.

Machine learning provides techniques, tools, and models that can aid in solving diagnostic and prognostic problems in a variety of clinical conditions. Machine learning algorithms are widely applied in classification of diseases based on ECG, EEG, and EMG signals [21]. Automated detection and classification of fever patterns using machine learning techniques with the specific algorithm-based classifier for specific diseases might have potential benefits such as increasing efficiency, reproducibility, and cost-effectiveness by providing early diagnosis of the disease and treatment, especially in undifferentiated fever cases.

It is through this study that we intend to record, analyze, and classify the tympanic temperature recordings of patients presenting with undifferentiated fever and using body temperature as a predictive variable for differentiating undifferentiated fevers.

## 2. Materials and Methods

### 2.1. Data Collection

This was an observational study conducted in a tertiary care hospital. A total of ninety-six (*n* = 96) patients presenting with prolonged fever symptom were recruited in the study. Patients who were on antipyretics, steroids, and with a history of hyperthermia and central nervous system disorder were excluded from the study. Malaria-infected fever patients were excluded in this study, because it is evident that malarial fever cycle occurs at every 48 hours [22] and we recorded the temperatures only for 24 hours. The patients were informed not to take a bath during temperature monitoring. Complete procedure of the study was explained to subjects before taking the informed consent and conducting the study. The study was approved by the institutional ethics committee. Anthropometric parameters like age, blood pressure, pulse rate, and BMI of each subject were noted. The continuous 24-hour tympanic temperature was recorded by using TherCom® temperature monitoring device [23, 24]. The final diagnosis of each patient was noted.

### 2.2. Preprocessing of Data

The temperature recordings were plotted and visually inspected for any missing data and filtered by using the Savitzky–Golay filter for smoothing the tracings without greatly distorting the signal. Each temperature recordings have 1440 data points, which were plotted at 9:00 AM to 9:00 AM timeframe.

### 2.3. Feature Extraction

Characteristic features of signal such as fast Fourier transform, entropy, energy, power, principal component analysis coefficients, autoregressive coefficients, wavelet transform coefficients, mean, and variance were extracted using MATLAB software (version R2013b), and visual observations of each temperature recordings such as presence of late night rise and presence of more than or equal to three peaks features were extracted. Extracted features were standardized using the normalization method. Further, 90% of extracted features were used for training and 10% for the test, using the classical 5-fold cross-validation setup.

To identify the accuracy of classification of the disease type, the four target diseases (tuberculosis, intracellular bacterial infections, dengue fever, and noninfectious (inflammatory and neoplastic) diseases) were assigned as responses and extracted features were assigned as predictors. Responses were assigned based on the final clinical diagnosis of each case corresponding to temperature recordings.

### 2.4. Evaluation of Algorithm

Evaluation of classification algorithm was done by using classification application in MATLAB, which has a set of algorithms, where we can train extracted feature datasets. The algorithm which gives the highest accuracy was selected. In our study, we found the highest classification accuracy in quadratic support vector machine (SVM) algorithm.

### 2.5. Statistical Analysis

Data were expressed as mean ± SD. Descriptive data analysis was done and an agreement between the classification of fever patterns by quadratic support vector machine learning algorithm and final diagnosis of the cases was performed by Kappa statistics by using Statistical Package for Social Sciences (SPSS) version 16, Chicago, IL. Feature extraction and area under receiver operating characteristic (ROC) curve of each categorized data were performed using the MATLAB software (version R2013b, the Mathworks, USA).

## 3. Results

A total of ninety-six (*n* = 96) patients presenting with undifferentiated fever were recruited in the study. As per the physician's diagnosis and based on laboratory diagnostic tests, subjects were categorized into tuberculosis (*n* = 28), intracellular bacterial infections (*n* = 27), dengue fever (*n* = 15), and noninfectious diseases (*n* = 26).  Table 1 summarizes the demographic details of each disease category. Demographic measures such as mean age, body mass index (BMI), blood pressure, and pulse rate did not differ between different disease groups.

We analyzed a quadratic support vector machine algorithm model for the differentiation of cases of the fever with 24-hour continuous tympanic temperature data and found an overall 71.9% accuracy in the algorithm. The algorithm performance for classifying the undifferentiated fever cases is summarized in Table 2. The overall area under ROC curve of each categorized data set is described in Table 3. The positive and negative predictive values and likelihood ratios of each categorized data set are described in Table 4. In summary, the quadratic support vector machine algorithm shows clinically significant accuracy in classifying assigned diseases.

We performed kappa agreement test between the classification of temperature patterns by quadratic support vector machine learning algorithm and with an actual diagnosis of cases.

We found a statistically significant good kappa agreement of 0.618 [*p* < 0.001, 95% CI (0.498–0.737)] between the quadratic support vector machine (SVM) learning algorithm and final diagnosis of cases.

## 4. Discussion

In this study, we found a very high yield in the quadratic support vector machine (SVM) learning algorithm in classifying undifferentiated fevers using data obtained from 24-hour continuous noninvasive temperature monitoring. We found that classification of undifferentiated fevers into four major categories is possible and is likely to optimize the evaluation of undifferentiated tropical fevers.

Undifferentiated tropical fevers are very perplexing issues for the internist or general physicians in resource-limited settings, because undirected investigations add to the cause and lead to inappropriate clinical decisions. The classification model confirmed the utility of body temperature signal as a primary variable for classifying the undifferentiated fevers. In particular, diagnostic yield for tuberculosis was extremely high and sensitivity and specificity of tuberculosis group were found to be 96.43% (81.65%–99.9%) and 91.18% (81.78%–96.6%), respectively (Table 3). This could help in limiting unnecessary investigations focusing on a group of diseases and will allow a targeted investigative approach in undifferentiated fevers.

We found that the SVM learning algorithm showed higher sensitivity of 96.43% (95%CI, 81.65–99.91) and specificity of 91.18% (81.78–96.6) in detecting tuberculosis in comparison to acid-fast bacilli smear test with a sensitivity of 67.5% (95%CI, 60.6–73.9) and specificity of 97.5% (95%CI, 97.0–97.9) among 5336 samples reported by Mathew et al. [25]. The SVM learning algorithm showed low sensitivity [53.33% (95%CI, 26.59–78.73)] and specificity [93.83% (95%CI, 86.18–97.97)] in predicting the cases of dengue in comparison to the sensitivity [77.3% (95%CI, 69.8–83.6)] and specificity [100% (95%CI, 98.5)] of the NS1 Ag rapid strip test for the diagnosis of dengue fever in 154 patients [26]. In case of intracellular bacterial infections, SVM learning algorithm presented sensitivity [55.56% (95% CI, 35.33–74.52)] and specificity [92.75% (95%CI, 83.89–97.61)] in predicting the bacterial infections from undifferentiated fever cases using features of temperature tracings which were comparable with findings of procalcitonin as a biomarker for bacterial infection with 64.5% sensitivity and 84.0% specificity in differentiating the bacterial infections from febrile patients as reported by Qu et al. [27]. The advantage of SVM learning algorithm is that one test is sufficient to differentiate four major clinical conditions, whereas culture or serology tests are to be performed separately for each clinical condition and these tests are invasive and expensive.

The procedure is simple, noninvasive, inexpensive, and reliable. The algorithm can easily be exported to any conventional computational devices, thereby allowing this to be implemented as a point of care diagnostic test. In addition, the 24-hour continuous temperature recording also helps us in identifying the undetected fever spikes in conventional monitoring method. Two scientific studies were reported on the significance of continuous temperature monitoring over conventional temperature monitoring method [4, 24]. Varela et al. studied in 62 patients presenting with fever and found that continuous temperature recording method detected mean of 0.7 (95% CI, 0.27–1.33) peaks of fever unnoticed by conventional care [4]. In our previous study, we found that intermittent nature of fever patterns was clearly detected by continuous recordings, whereas conventional method failed to capture 29.9% of intermittent nature of fever patterns. Hence, capturing complete variations of body temperature was an added benefit of 24-hour continuous temperature monitoring method.

In the previous study, some of the mathematical models were utilized for prediction and prognostication of certain clinical conditions. In two different studies, Varela et al. applied approximate entropy alone, and along with detrended fluctuation analysis (DFA) to measure the complexity of temperature curve in correlating with SOFA values for predicting survival in critically ill patients [17, 18]. Papaioannou et al. assessed the temperature complexity in a cohort of critically ill patients who developed sepsis and septic shock during their stay in ICU and found an early prediction of mortality in them by extracting Tsallis entropy (TsEn) and Shannon entropy (Sh) as features [28]. Varela et al. also tried the classification of diagnostic groups using complexity variable (approximate entropy) [4]. However, researchers did not yield fruitful results probably because of single or either of the two mathematical parameters such as approximate entropy and DFA, TsEn, and Shannon entropy were looked for in the signal, and the other features which we believe are important were not evaluated. Moreover, the previous studies addressed the complexity of temperature signal in critical care patients and not in formal settings. Wavelet analysis and multiscale entropy were used in one study by Papaioannou et al. [15]; however, in our study, we included wavelet coefficients as a feature of one-dimensional signal and applied it in the machine learning processes. In addition to this, we observed some of the important features visually. We combined both visually important and other extracted features in the machine learning algorithm, which appears to yield a very high success rate in appropriate classification.

While the concept of continuous fever recording began way back a century ago, [11], somehow it was not taken forward because of hardware and software impediments. Now, there is a need to revisit this interesting concept with manifold better hardware and software technologies and their issues have been largely dealt with.

Limitation of the study includes relatively very small sample size, but case mixes were similar those in other reported study [29]. Some technical difficulties while monitoring temperature were mainly the falling off of the tympanic probe from the ear canal which interrupts the continuous recording. Secondly, the loose connection of probe to the data logger interrupted continuous data storage. This was also mentioned in a previously conducted study by Varela et al. [4]. We applied the Savitzky–Golay filter for noise reduction and smoothening of the temperature signal without distorting the signal. There are other filtering methods that can be applied to filter the data which may increase the yield in classification algorithm.

The interesting observations found in this small group of samples need to be studied in a bigger sample size. Once the large data sets are obtained, artificial neural network analysis may offer a higher yield. Extensive use of this promising algorithm may yield significant output with a larger dataset in the future, which will further allow us to apply artificial neuronal network at that point in time. We have done the analysis in undifferentiated fever settings, but it is very likely that it may also be useful in pyrexia of unknown origin settings. We have four classifying groups of samples, but with the expanding samples, further groups may be apparently evident and might improve the accuracy of the model. Another important possibility would be to record two or three days of temperature and to look for patterns and extract features in a bigger recording time frame.

## 5. Conclusion

Use of supervised automated classifying algorithm can provide a significant clue for the discrimination of undifferentiated fevers at an early stage. An accurate diagnostic test aids the process of quick decision-making by the physician, in addition to minimizing the cost of unnecessary diagnostic tests. As a noninvasive tool, temperature pattern classifier algorithm may become an essential, additional diagnostic tool which can be used as inpatient and outpatient clinical settings in the future for the evaluation of fever of various clinical conditions.

## Figures and Tables

**Table 1:** Demographic details of subjects.

Sl number	Cases (*N* = 96)	Age, mean (SD), years	BMI, mean (SD), kg/M^2^	Blood pressure		Pulse rate, mean (SD), per min
				SBP, mean (SD), mmHg	DBP, mean (SD), mmHg	
1	Tuberculosis (*N* = 28)	44.14 (14.39)	20.07 (3.61)	121.07 (11.0)	79.71 (7.45)	83.25 (10.24)
2	Intracellular bacterial infections (*N* = 27)	32.18 (13.77)	23.10 (3.53)	124.11 (9.33)	80.0 (3.92)	82.51 (5.36)
3	Dengue fever (*N* = 15)	41.13 (12.50)	24.10 (5.52)	122.00 (9.41)	78.93 (5.49)	81.33 (7.15)
4	Noninfectious diseases (*N* = 26)	44.03 (15.05)	22.03 (3.46)	123.00 (10.52)	78.65 (7.42)	83.38 (8.46)

BMI: body mass index; SBP: systolic blood pressure; DBP: diastolic blood pressure.

**Table 2:** Confusion matrix of quadratic support vector machine algorithm of undifferentiated fever cases.

Cases	Tuberculosis	Intracellular bacterial infections	Dengue fever	Noninfectious diseases
Tuberculosis	27	01	0	0
Intracellular bacterial infections	06	15	01	05
Dengue fever	0	01	08	06
Noninfectious diseases	0	03	04	19

**Table 3:** Area under ROC curve of quadratic support vector machine algorithm.

Cases	AUROC^#^	False-positive rate	True-positive rate	Sensitivity (%)	Specificity (%)
Tuberculosis	0.961	0.088	0.964	96.43 (81.65–99.91)	91.18 (81.78–96.6)
Intracellular bacterial infections	0.801	0.072	0.555	55.56 (35.33–74.52)	92.75 (83.89–97.61)
Dengue fever	0.815	0.061	0.533	53.33 (26.59–78.73)	93.83 (86.18–97.97)
Noninfectious diseases	0.818	0.157	0.730	73.08 (52.21–88.43)	84.29 (73.62–91.89)

^#^Area under ROC curve was automatically calculated and given by MATLAB software.

**Table 4:** Positive and negative predictive values of quadratic support vector machine algorithm.

Cases	Positive predictive value (%)	Negative predictive value (%)	Positive likelihood ratio	Negative likelihood ratio
Tuberculosis	81.82 (67.63–90.65)	98.41 (90.03–99.77)	10.93 (5.07–23.54)	0.04 (0.01–0.27)
Intracellular bacterial infections	75.00 (54.72–88.16)	84.21 (77.68–89.10)	7.67 (3.09–19.03)	0.48 (0.31–0.73)
Dengue fever	61.54 (37.70–80.88)	91.57 (86.31–94.92)	8.64 (3.27–22.84)	0.50 (0.29–0.86)
Noninfectious diseases	63.33 (48.90–75.72)	89.39 (81.61–94.12)	4.65 (2.58–8.39)	0.32 (0.17–0.61)

## References

[1] Joshi R. (2009). *The Epidemiologic Features of Acute Encephalitis Syndrome in Central India*.

[2] Susilawati T. N., McBride W. J. (2014). Acute undifferentiated fever in Asia: a review of the literature. *Southeast Asian Journal of Tropical Medicine and Public Health*.

[3] Joshi R., Kalantri S. P. (2015). Acute undifferentiated fever: management algorithm. *Update on Tropical Fever*.

[4] Varela M., Ruiz-Esteban R., Martinez-Nicolas A., Cuervo-Arango J. A., Barros C., Delgado E. G. (2011). “Catching the spike and tracking the flow”: Holter-temperature monitoring in patients admitted in a general internal medicine ward. *International Journal of Clinical Practice*.

[5] Vincent J.-L., Weil M. H. (2006). Fluid challenge revisited. *Critical Care Medicine*.

[6] Clarke S., Parris R., Reynard K. (2005). Core–peripheral temperature gradient as a diagnostic test in dyspnoea. *Emergency Medicine Journal*.

[7] Kiekkas P., Velissaris D., Karanikolas M. (2010). Peak body temperature predicts mortality in critically ill patients without cerebral damage. *Heart & Lung: The Journal of Acute and Critical Care*.

[8] Kaplan L. J., Frangos S. (2005). Clinical review: acid–base abnormalities in the intensive care unit-part II. *Critical Care*.

[9] Menon V., Slater J. N., White H. D., Sleeper L. A., Cocke T., Hochman J. S. (2000). Acute myocardial infarction complicated by systemic hypoperfusion without hypotension: report of the SHOCK trial registry. *The American Journal of Medicine*.

[10] Joly H. R., Weil M. H. (1969). Temperature of the great toe as an indication of the severity of shock. *Circulation*.

[11] Woodhead G., Varrier-Jones P. (1921). The value of the quasi-continuous temperature record in the early diagnosis and prognosis of tuberculosis. *The Lancet*.

[12] Varela M., Jimenez L., Farina R. (2003). Complexity analysis of the temperature curve: new information from body temperature. *European Journal of Applied Physiology*.

[13] Varela M., Churruca J., Gonzalez A., Martin A., Ode J., Galdos P. (2006). Temperature curve complexity predicts survival in critically ill patients. *American Journal of Respiratory and Critical Care Medicine*.

[14] Pikkujamsa S. M., Makikallio T. H., Sourander L. B. (1999). Cardiac interbeat interval dynamics from childhood to senescence: comparison of conventional and new measures based on fractals and chaos theory. *Circulation*.

[15] Papaioannou V. E., Chouvarda I. G., Maglaveras N. K., Pneumatikos I. A. (2012). Temperature variability analysis using wavelets and multiscale entropy in patients with systemic inflammatory response syndrome, sepsis, and septic shock. *Critical Care*.

[16] Drewry A. M., Fuller B. M., Bailey T. C., Hotchkiss R. S. (2013). Body temperature patterns as a predictor of hospital-acquired sepsis in afebrile adult intensive care unit patients: a case-control study. *Critical Care*.

[17] Varela M., Calvo M., Chana M., Gomez-Mestre I., Asensio R., Galdos P. (2005). Clinical implications of temperature curve complexity in critically ill patients. *Critical Care Medicine*.

[18] Cuesta D., Varela M., Miro P. (2007). Predicting survival in critical patients by use of body temperature regularity measurement based on approximate entropy. *Medical & Biological Engineering & Computing*.

[19] Fernandez E., Smieja M., Walter S. D., Loeb M. (2016). A predictive model to differentiate dengue from other febrile illness. *BMC Infectious Diseases*.

[20] Tanner L., Schreiber M., Low J. G. (2008). Decision tree algorithms predict the diagnosis and outcome of dengue fever in the early phase of illness. *PLoS Neglected Tropical Diseases*.

[21] Shoeb A. H., Guttag J. V. Application of machine learning to epileptic seizure detection.

[22] Ogoina D. (2011). Fever, fever patterns and diseases called “fever”—a review. *Journal of Infection and Public Health*.

[23] Cuesta-Frau D., Varela M., Aboy M., Miro-Martinez P. (2009). Description of a portable wireless device for high-frequency body temperature acquisition and analysis. *Sensors*.

[24] Dakappa P. H., Bhat G. K., Bolumbu G., Rao S. B., Adappa S., Mahabala C. (2016). Comparison of conventional mercury thermometer and continuous TherCom® temperature recording in hospitalized patients. *Journal of Clinical and Diagnostic Research*.

[25] Mathew P., Kuo Y. H., Vazirani B., Eng R. H., Weinstein M. P. (2002). Are three sputum acid-fast bacillus smears necessary for discontinuing tuberculosis isolation?. *Journal of Clinical Microbiology*.

[26] Chaterji S., Allen J. C., Chow A., Leo Y. S., Ooi E. E. (2011). Evaluation of the NS1 rapid test and the WHO dengue classification schemes for use as bedside diagnosis of acute dengue fever in adults. *The American Journal of Tropical Medicine and Hygiene*.

[27] Qu J., Lü X., Liu Y., Wang X. (2015). Evaluation of procalcitonin, C-reactive protein, interleukin-6 & serum amyloid A as diagnostic biomarkers of bacterial infection in febrile patients. *Indian Journal of Medical Research*.

[28] Papaioannou V. E., Chouvarda I. G., Maglaveras N. K., Baltopoulos G. I., Pneumatikos I. A. (2013). Temperature multiscale entropy analysis: a promising marker for early prediction of mortality in septic patients. *Physiological Measurement*.

[29] Abrahamsen S. K., Haugen C. N., Rupali P. (2013). Fever in the tropics: aetiology and case-fatality-a prospective observational study in a tertiary care hospital in South India. *BMC Infectious Diseases*.

